# Analysis by region of outcomes for patients with advanced renal cell carcinoma treated with cabozantinib or everolimus: a sub-analysis of the METEOR study

**DOI:** 10.1080/0284186X.2021.1995041

**Published:** 2021-11-04

**Authors:** Manuela Schmidinger, Robert J. Motzer, Frederic Rolland, Michael Staehler, Michael Rink, Margitta Retz, Tibor Csoszi, John A. McCaffrey, Ugo De Giorgi, Claudia Caserta, Ignacio Duran, Fawzi Benzaghou, Douglas O. Clary, Laurence Albiges, Toni K. Choueiri, Nizar M. Tannir

**Affiliations:** a Department of Urology, Medical University of Vienna, Vienna, Austria; b Memorial Sloan Kettering Cancer Center, New York, NY, USA; c Institut de Cancérologie de l’Ouest, Saint-Herblain, France; d Department of Urology, Ludwig-Maximilians-Universität München, Munich, Germany; e Department of Urology, University Medical Center Hamburg-Eppendorf, Hamburg, Germany; f Rechts der Isar Medical Center, Technical University of Munich, Munich, Germany; g Jász-Nagykun-Szolnok County Hospital, Szolnok, Hungary; h Cancer Trials Ireland, Dublin, Ireland; i IRCCS Istituto Romagnolo per lo Studio dei Tumori (IRST) “Dino Amadori”, Meldola, Italy; j Medical and Translational Oncology Unit, Azienda Ospedaliera Santa Maria, Terni, Italy; k Medical Oncology Department, Hospital Universitario Marques de Valdecilla (IDIVAL), Santander, Spain; l Ipsen Bioscience, Oncology R&D, Cambridge, MA, USA; m Exelixis Inc., Alameda, CA, USA; n Medical Oncology, Gustave Roussy, Université Paris-Saclay, Villejuif, France; o Dana-Farber Cancer Institute, Boston, MA, USA; p MD Anderson Cancer Center Hospital, The University of Texas, Houston, TX, USA

**Keywords:** Cabozantinib, everolimus, renal cell carcinoma, METEOR regional sub-analysis

## Abstract

**Introduction:**

METEOR was a phase 3 trial (NCT01865747) of cabozantinib versus everolimus in adults with advanced or metastatic clear cell RCC previously treated with VEGF receptor (VEGFR) tyrosine kinase inhibitors (TKIs). This *post hoc* analysis of METEOR compared outcomes for patients recruited from European and non-European countries.

**Material and methods:**

Adults with advanced/metastatic clear cell RCC who had received ≥ 1 prior VEGFR-TKI treatment were randomized 1:1 to receive cabozantinib or everolimus. Patients were categorized by recruitment region: Europe or outside of Europe (rest of world [RoW]). Progression-free survival (PFS), overall survival (OS), objective response rate (ORR), and adverse events (AEs) were compared between regional subgroups.

**Results:**

In total, there were 320 eligible patients from Europe (cabozantinib, 167; everolimus, 153) and 338 from RoW (North America, 240 patients; Asia-Pacific, 86; Latin America, 12; randomized as cabozantinib, 163; everolimus, 175). PFS and OS were longer with cabozantinib than with everolimus and similar for the Europe and RoW subgroups. For PFS, the hazard ratio (HR) for cabozantinib versus everolimus was 0.54 for the Europe subgroup (*p* < .001) and 0.50 for the RoW subgroup (*p* < .001). For OS, the HR was 0.75 for the Europe subgroup (*p* = .034) and 0.69 for the RoW subgroup (*p* = .006). ORR in the Europe subgroup was 15% for cabozantinib and 3.9% for everolimus (*p* < .001). For the RoW subgroup, ORR was 20% for cabozantinib and 2.9% for everolimus (*p* < .001). Incidence of grade 3/4 AEs were similar for the Europe (cabozantinib, 74%; everolimus, 58%) and RoW subgroups (cabozantinib, 69%; everolimus, 64%).

**Conclusion:**

In the METEOR trial, efficacy outcomes for patients recruited from European and non-European countries favored cabozantinib over everolimus. The efficacy and safety results for the regional subgroups were consistent with those of the overall METEOR population.

## Introduction

In 2018, over 400,000 people worldwide received a diagnosis of renal cancer and approximately 175,000 deaths were attributed to this disease (representing 1.8% of all cancer-related deaths) [1]. Renal cell carcinoma (RCC) accounts for approximately 80% of renal cancers [2], with clear cell carcinoma the most common histological subtype, reported in 75–80% of cases [3].

The von Hippel–Lindau tumor suppressor gene is frequently inactivated in RCC, leading to overexpression of hypoxia-inducible factors and downstream targets, including AXL, MET, and vascular endothelial growth factor (VEGF) [4–6]. Novel targeted therapies, including VEGF-pathway inhibitors, have significantly improved patient outcomes since their introduction [7].

Cabozantinib, a tyrosine kinase inhibitor (TKI), has activity against multiple receptor tyrosine kinases, including AXL, MET, and the VEGF receptor (VEGFR) [4]. Cabozantinib is approved in the USA for adults with advanced RCC in both first and subsequent lines of treatment [8], in Europe for adults with treatment-naive advanced RCC with intermediate or poor risk and adults following prior VEGF-targeted therapy [9], and in both the USA and Europe as first-line treatment in combination with nivolumab. In the randomized phase 2 CABOSUN trial (NCT01835158) in treatment-naive adults with advanced or metastatic clear cell RCC with poor or intermediate risk, cabozantinib demonstrated significantly improved efficacy compared with sunitinib, based on median progression-free survival (PFS; 8.2 versus 5.6 months; adjusted hazard ratio [HR] for progression or death 0.66 [95% confidence interval (CI) 0.46–0.95]; one-sided *p* = .012) and objective response rate (ORR; 33% versus 12%, as assessed by investigator review) [10]. In the randomized phase, 3 METEOR trial (NCT01865747) in adults with advanced or metastatic clear cell RCC who had been previously treated with VEGFR-TKIs, cabozantinib demonstrated significantly improved efficacy compared with everolimus, based on median PFS (7.4 versus 3.9 months; HR 0.51 [95% CI 0.41–0.62]; *p* < .0001), ORR (17% versus 3%) and median overall survival (OS; 21.4 versus 16.5 months) [11,12].

METEOR was conducted in 26 countries [11]. To establish whether the findings from the overall study population are reflected in patients from European and non-European countries, we conducted *post hoc* analyses of efficacy and safety outcomes from METEOR in these geographical subgroups.

## Materials and methods

### Study design and patients

METEOR study design and methods have been reported previously [11,12]. Briefly, participants were aged 18 years or older with advanced or metastatic clear cell RCC. Key inclusion criteria were: at least one prior VEGFR-TKI treatment; disease progression during or within the previous 6 months of the most recent VEGFR-TKI treatment; and a Karnofsky Performance Status score of at least 70%. Patients were randomized 1:1 to receive cabozantinib (60 mg once daily) or everolimus (10 mg once daily). Dose reductions for cabozantinib (40 mg, then 20 mg) and everolimus (5 mg, then 2.5 mg) were permitted for managing adverse events (AEs). The study protocol was approved by the ethics committee or institutional review board at each participating center, and all patients provided written informed consent.

### Assessments

The primary endpoint in the METEOR study was PFS, and secondary endpoints included OS and ORR [11,12]. Tumor response and progression were assessed by an independent radiology committee according to Response Evaluation Criteria in Solid Tumors version 1.1 criteria [13,14] at screening, every 8 weeks for the first year, and every 12 weeks thereafter. Reported AEs were graded according to National Cancer Institute Common Terminology Criteria for Adverse Events version 4.0 [15] and coded using Medical Dictionary for Regulatory Activities version 17.0.

### Statistical analyses

Statistical methods used for the primary analysis of METEOR trial data are reported elsewhere [11,12]. Efficacy was evaluated in the intention-to-treat (ITT) population (defined as all randomized patients), and safety was assessed in patients who had received study treatment. The current analyses were performed *post hoc* and were not powered to detect statistically significant differences between groups.

Patients from the METEOR trial were categorized according to whether they were recruited from Europe or outside of Europe (North America [Canada and the USA], the Asia-Pacific region [Taiwan, South Korea, and Australia], and Latin America), hereafter referred to as rest of the world (RoW).

Efficacy (PFS, OS, and ORR) and safety (AEs) outcomes and anticancer treatments, particularly VEGFR-TKIs, taken before or after study treatment were assessed.

PFS and OS were evaluated using Kaplan–Meier analysis. The current nominal statistical comparisons of PFS and OS for cabozantinib versus everolimus in each geographical region were made using unstratified HRs with 95% CIs and estimated using a Cox regression model.

ORRs were compared between treatment groups for each geographical region using stratified Cochran–Mantel–Haenszel tests and unstratified chi-squared tests. All other comparisons are descriptive. The data cutoff date for the outcomes assessed in these *post hoc* analyses was October 2, 2016.

## Results

### Patient demographics and clinical characteristics

Of 658 patients randomly assigned to receive cabozantinib or everolimus (ITT population), 320 (48.6%) were from Europe and 338 (51.4%) from the RoW. Within the RoW subgroup, 240 patients (71.0%) were from North America, 86 (25.4%) were from the Asia-Pacific region and 12 (3.6%) were from Latin America. In the Europe and RoW subgroups, respectively, 167 and 164 patients were treated with cabozantinib, and 151 and 171 patients were treated with everolimus (safety population).

Baseline characteristics and demographics were generally balanced between groups (Table 1). Median ages were in the range 61–63 years, and most participants were male and white. Compared with the European subgroup, the RoW subgroup included higher proportions of patients identifying as Asian or Black/African American, but numbers were still low. Most participants in all groups (68.3–74.2%) had received one VEGFR-TKI before the study, most commonly sunitinib (56.4–70.7%) (Table 1).

### Efficacy

**Figure 1.**
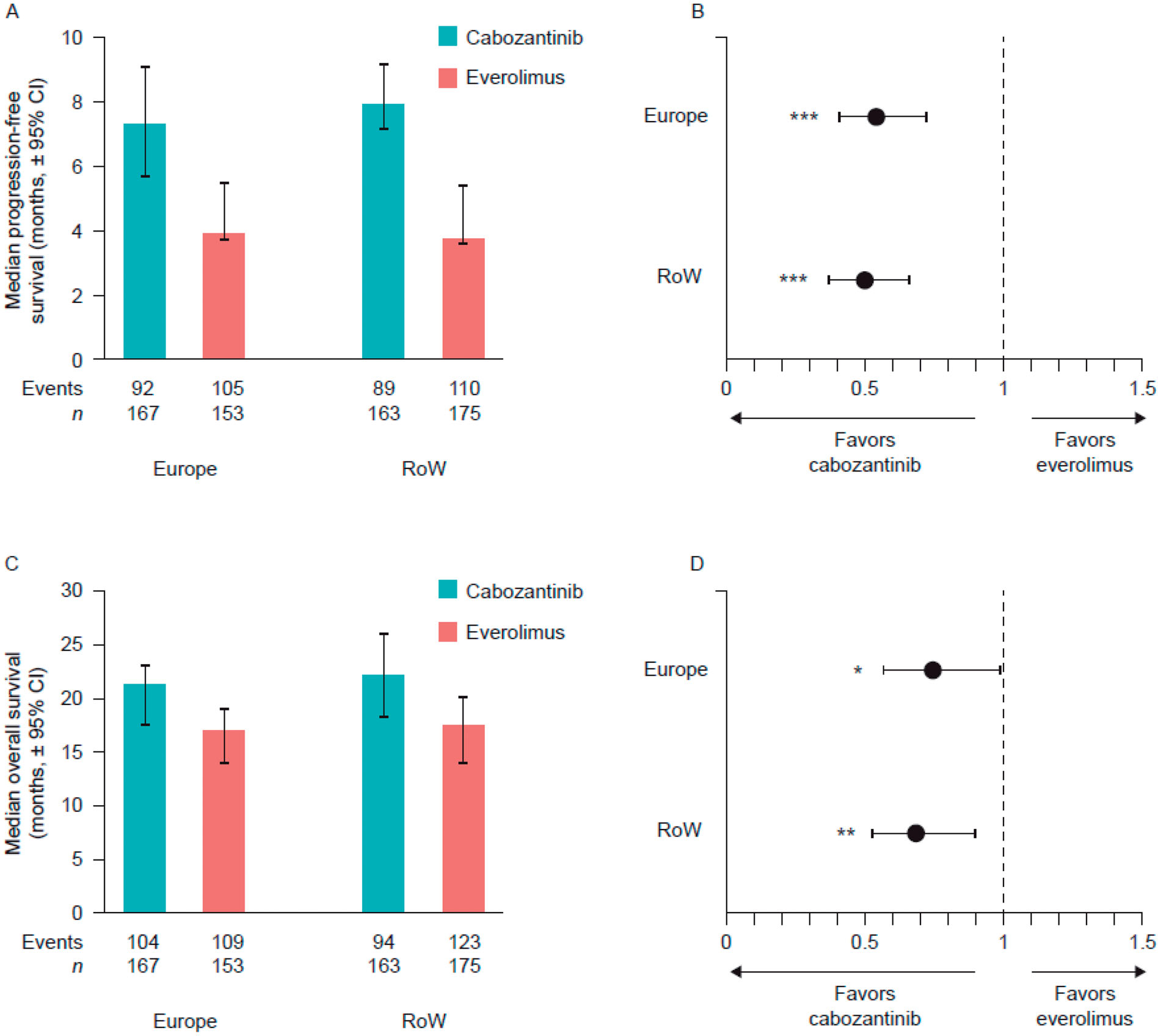
Progression-free survival and overall survival outcomes in the Europe and rest of the world subgroups of the METEOR study. Median progression-free survival time (A) and cabozantinib versus everolimus hazard ratios (B). Overall survival time (C) and cabozantinib versus everolimus hazard ratios (D). *p < .05; **p < .01; ***p < .001. CI: confidence interval; RoW: rest of the world.

Median PFS and OS in patients who received cabozantinib or everolimus (ITT population) were similar in both subgroups (Figure 1). Among patients who received cabozantinib, in the Europe and RoW subgroups, respectively, median (95% CI) PFS was 7.3 (5.7–9.1) months and 7.9 (7.2–9.2) months, while median (95% CI) OS was 21.1 (17.4–23.0) months and 22.0 (95% CI 18.2–26.0) months. In the everolimus treatment groups, median (95% CI) PFS and OS were 3.9 (3.7–5.5) months and 16.9 (14.0–19.0) months, respectively, in the Europe group, and 3.7 (3.6–5.4) months and 17.3 (13.8–20.1) months, respectively, in the RoW group.

In both subgroups, PFS and OS were longer in patients who received cabozantinib than in those who received everolimus (Figure 1). For PFS, the HR (95% CI) was 0.54 (0.41–0.72; *p* < .001) in the Europe group and 0.50 (0.37–0.66; *p* < .001) in the RoW group. For OS, the HR (95% CI) was 0.75 (0.57–0.98; *p* = .034) in the Europe group and 0.69 (0.52–0.90; *p* = .006) in the RoW group.

The ORR, as assessed by independent radiology review, was higher in patients treated with cabozantinib than in those treated with everolimus in both subgroups. In the Europe group, the ORR (95% CI) was 15% (10–21%) for cabozantinib and 3.9% (1–8%) for everolimus (*p* < .001). In the RoW group, the ORR (95% CI) was 20% (14–27%) for cabozantinib and 2.9% (0.9–7%) for everolimus (*p* < .001). All confirmed responses were partial (Supplementary Table 1).

### Safety

In the safety population, the incidence of treatment-emergent AEs was at least 99% in any group and, for most patients (89–99%), treatment-emergent AEs were judged to be related to treatment. The proportion of patients experiencing serious treatment-related treatment-emergent AEs was similar across the subgroups (range 12–19%) (Supplementary Table 2).

The proportions of patients who received cabozantinib and everolimus, respectively, who experienced all-cause grade 3/4 AEs were similar in the Europe subgroup (74% and 58%) and the RoW subgroup (69% and 64%). In the Europe and RoW subgroups, the most frequently reported (in 10% or more of patients) grade 3/4 AEs were hypertension (18% and 13%, respectively), diarrhea (14% and 13%, respectively), and fatigue (11% each) with cabozantinib, and anemia (18% and 16%, respectively) with everolimus.

### Subsequent anticancer treatments

In the Europe and RoW subgroups, respectively, 64 patients (38%) and 63 patients (39%) treated with cabozantinib and 69 patients (45%) and 86 patients (49%) were treated with everolimus received subsequent anticancer therapy (Supplementary Table 3).

## Discussion

The METEOR trial compared the efficacy and safety profile of cabozantinib with that of everolimus in patients with advanced or metastatic RCC who had progressed after VEGFR-TKI therapy [11,12]. Findings from these *post hoc* analyses of data from METEOR were consistent with those reported for the overall study population [11]. In subgroups of patients recruited from European or non-European (North America, the Asia-Pacific region, and Latin America) regions, cabozantinib treatment was associated with improved efficacy compared with everolimus treatment.

Baseline demographic and clinical characteristics were generally well balanced between the treatment groups in the overall METEOR population [11]. However, this large study was conducted in 26 countries [11,12]. Given that participant characteristics that can influence study outcomes (such as ethnicity, socioeconomic makeup, healthcare systems, and access to treatments or subsequent therapies [16–21]) may vary by geographic region, we evaluated efficacy and safety outcomes from the METEOR study in subgroups of patients from Europe and the RoW.

In the present *post hoc* analyses, efficacy outcomes with cabozantinib or everolimus were consistent between the two subgroups, and with those of the overall METEOR study population [12]. The uptake of subsequent anticancer therapies was also balanced between these subgroups. Likewise, safety profiles for cabozantinib and everolimus were generally similar in both subgroups, with no new safety signals, and consistent with those of the overall METEOR study population [12]. Proportions of patients assigned to cabozantinib or everolimus who reported grade 3/4 AEs were similar in the Europe subgroup (74% and 58%, respectively) and RoW subgroup (69% and 64%, respectively), and consistent with those of the overall METEOR study population (71% and 60%, respectively) [12]. Hypertension was the most common grade 3/4 AE with cabozantinib and anemia with everolimus. This was consistent between the two subgroups and with the overall METEOR study population [12].

The absence of major differences in efficacy and safety outcomes between the two subgroups may reflect the fact that baseline patient demographic and clinical characteristics were generally well balanced. There were also no major differences in prior VEGFR-TKI treatment, although a previous sub-analysis of METEOR study data found that prior therapy had no substantial effect on clinical outcomes with cabozantinib [22].

Various studies have reported interethnic differences, particularly between Asian and non-Asian patients, in exposure, efficacy, and safety of TKIs used in cancer treatment [16–18]. Although the RoW subgroup in the present study included a higher proportion of Asian patients than the Europe subgroup, the absolute number of Asian patients was small relative to the overall population, and no marked differences in efficacy or safety outcomes were observed between the two subgroups.

An important limitation of the present analyses was the incorporation of participants from North America, the Asia-Pacific region, and Latin America into a single subgroup, thereby losing some of the regional granularity that would have resulted from considering these regions separately. However, as previously reported [12], in the overall study population, the proportions of patients who received cabozantinib and of those who received everolimus who were recruited from Latin America (2% each) and the Asia-Pacific region (12% and 14%, respectively) were relatively small. Furthermore, both subgroups were numerically well balanced, with each including approximately half of the participants. A previous *post hoc* analysis of the METEOR study presented at the European Society for Medical Oncology 2016 Congress reported outcomes for patients from Europe, North America, and the Asia-Pacific region, despite the small sample sizes involved, and demonstrated that improvements in PFS, OS, and ORR were consistent across these subgroups [23].

In conclusion, these *post hoc* analyses of data from the METEOR study of cabozantinib compared with everolimus, in patients with advanced or metastatic RCC who had received previous treatment with VEGFR-TKIs, indicate that efficacy and safety outcomes were similar in the Europe and RoW subgroups and consistent with those observed in the overall METEOR study population. In both regional subgroups, PFS, OS, and ORR favored cabozantinib over everolimus. These data provide confidence that efficacy and safety outcomes observed in the multinational METEOR study are reflected in both European and non-European populations, and further support cabozantinib as a preferred second-line treatment option for patients with RCC in this setting [24].

## Supplementary Material

Supplementary Material

## Figures and Tables

**Table 1.** Baseline demographics and clinical characteristics of participants in the Europe and rest of the world subgroups of the METEOR study.

	Europe		Rest of the world	
Characteristic	Cabozantinib (*n* = 167)	Everolimus (*n* = 153)	Cabozantinib (*n* = 163)	Everolimus (*n* = 175)

Median age (range), years	63.0 (32–86)	63.0 (33–84)	61.0 (36–83)	61.0 (31 –84)
Male, *n* (%)	122 (73)	114 (75)	131 (80)	126 (72)
Geographic region, *n* (%)				
Europe	167 (100)	153 (100)	0	0
North America	0	0	118 (72)	122 (70)
Asia-Pacific	0	0	39 (24)	47 (27)
Latin America	0	0	6 (3.7)	6 (3.4)
Race, *n* (%)				
White	139 (83)	122 (80)	130 (80)	141 (81)
Black or African American	0	0	6 (3.7)	3 (1.7)
Asian	1 (0.6)	1 (0.7)	20 (12)	25 (14)
Other	13 (7.8)	10 (6.5)	6 (3.7)	3 (1.7)
Not reported	14 (8.4)	20 (13)	1 (0.6)	2 (1.1)
Missing	0	0	0	1 (0.6)
Number of prior VEGFR-TKI therapies, *n* (%)				
1	114 (68.3)	108 (70.6)	121 (74.2)	121 (69.1)
2	49 (29.3)	43 (28.1)	35 (21.5)	48 (27.4)
≥ 3	4 (2.4)	2 (1.3)	7 (4.3)	6 (3.4)
Type of prior VEGFR-TKI therapy, *n* (%)				
Axitinib	26 (15.6)	28 (18.3)	26 (16.0)	27 (15.4)
Pazopanib	59 (35.3)	54 (35.3)	85 (52.1)	82 (46.9)
Sorafenib	14 (8.4)	12 (7.8)	7 (4.3)	19 (10.9)
Sunitinib	118 (70.7)	101 (66.0)	92 (56.4)	104 (59.4)
Other VEGFR-TKI	7 (4.2)	6 (3.9)	3 (1.8)	3 (1.7)

TKI: tyrosine kinase inhibitor; VEGFR: vascular endothelial growth factor receptor.

## References

[1] Bray F, Ferlay J, Soerjomataram I, Global cancer statistics 2018: GLOBOCAN estimates of incidence and mortality worldwide for 36 cancers in 185 countries. CA Cancer J Clin. 2018;68(6): 394–424. 30207593 10.3322/caac.21492

[2] Escudier B, Porta C, Schmidinger M, Renal cell carcinoma: ESMO clinical practice guidelines for diagnosis, treatment and follow-up. Ann Oncol. 2016;27(suppl 5):v58–v68. 27664262 10.1093/annonc/mdw328

[3] Cairns P Renal cell carcinoma. Cancer Biomark. 2010;9(1–6): 461–473. 22112490 10.3233/CBM-2011-0176 PMC3308682

[4] Atkins MB, Tannir NM. Current and emerging therapies for first-line treatment of metastatic clear cell renal cell carcinoma. Cancer Treat Rev. 2018;70:127–137. 30173085 10.1016/j.ctrv.2018.07.009

[5] Dutcher JP. Update on the biology and management of renal cell carcinoma. J Investig Med. 2019;67(1):1–10. 10.1136/jim-2018-000918 30455223

[6] Potemski P, Szczylik C, Tomczak P, Cabozantinib for the treatment of renal cell carcinoma patients. Oncol Clin Pract. 2017; 13:147–155.

[7] Tsao CK, Liaw B, He C, Moving beyond vascular endothelial growth factor-targeted therapy in renal cell cancer: latest evidence and therapeutic implications. Ther Adv Med Oncol. 2017; 9(4):287–298. 28491148 10.1177/1758834016687261 PMC5405995

[8] U.S. Food and Drug Administration. Cabozantinib prescribing information; 2012. [updated January 2021; cited 2021 May 28]. Available from: https://www.accessdata.fda.gov/drugsatfda_docs/label/2021/208692s010lbl.pdf

[9] European Medicines Agency. Cabometyx: EPAR - Product Information; 2020. [updated 2021 May 5; cited 2021 May 8]. Available from: https://www.ema.europa.eu/en/documents/product-information/cabometyx-epar-product-information_en.pdf

[10] Choueiri TK, Halabi S, Sanford BL, Cabozantinib versus sunitinib as initial targeted therapy for patients with metastatic renal cell carcinoma of poor or intermediate risk: the alliance A031203 CABOSUN trial. J Clin Oncol. 2017;35(6):591–597. 28199818 10.1200/JCO.2016.70.7398 PMC5455807

[11] Choueiri TK, Escudier B, Powles T, Cabozantinib versus everolimus in advanced renal-cell carcinoma. N Engl J Med. 2015; 373(19):1814–1823. 26406150 10.1056/NEJMoa1510016 PMC5024539

[12] Choueiri TK, Escudier B, Powles T, Cabozantinib versus everolimus in advanced renal cell carcinoma (METEOR): final results from a randomised, open-label, phase 3 trial. Lancet Oncol. 2016; 17(7):917–927. 27279544 10.1016/S1470-2045(16)30107-3

[13] Schwartz LH, Litière S, De Vries E, RECIST 1.1-Update and clarification: from the RECIST committee. Eur J Cancer. 2016;62: 132–137. 27189322 10.1016/j.ejca.2016.03.081 PMC5737828

[14] Eisenhauer EA, Therasse P, Bogaerts J, New response evaluation criteria in solid tumours: revised RECIST guideline (version 1.1). Eur J Cancer. 2009;45(2):228–247. 19097774 10.1016/j.ejca.2008.10.026

[15] National Cancer Institute. Common Terminology Criteria for Adverse Events (CTCAE) v.4; 2010. [updated 2010 June 14; cited 2021 May 8]. Available from: http://evs.nci.nih.gov/ftp1/CTCAE/About.html

[16] Touma JA, Mclachlan AJ, Gross AS. The role of ethnicity in personalized dosing of small molecule tyrosine kinase inhibitors used in oncology. Transl Cancer Res. 2017;6(S10):S1558–S1591.

[17] Liu X, Fiocco M, Swen JJ, Assessment of ethnic differences in sunitinib outcome between Caucasian and Asian patients with metastatic renal cell carcinoma: a meta-analysis. Acta Oncol. 2017;56(4):582–589. 27924664 10.1080/0284186X.2016.1265666

[18] Guo J, Jin J, Oya M, Safety of pazopanib and sunitinib in treatment-naive patients with metastatic renal cell carcinoma: Asian versus non-Asian subgroup analysis of the COMPARZ trial. J Hematol Oncol. 2018;11(1):69. 29788981 10.1186/s13045-018-0617-1 PMC5964681

[19] Sims JN, Yedjou CG, Abugri D, Racial disparities and preventive measures to renal cell carcinoma. IJERPH. 2018;15(6):1089. 10.3390/ijerph15061089 PMC6024978 29843394

[20] Znaor A, Lortet-Tieulent J, Laversanne M, International variations and trends in renal cell carcinoma incidence and mortality. Eur Urol. 2015;67(3):519–530. 25449206 10.1016/j.eururo.2014.10.002

[21] Lopes GDL, de Souza JA, Barrios C. Access to cancer medications in low- and middle-income countries. Nat Rev Clin Oncol. 2013;10(6):314–322. 23568416 10.1038/nrclinonc.2013.55

[22] Powles T, Motzer RJ, Escudier B, Outcomes based on prior therapy in the phase 3 METEOR trial of cabozantinib versus everolimus in advanced renal cell carcinoma. Br J Cancer. 2018;119(6): 663–669. 30197417 10.1038/s41416-018-0164-0 PMC6173766

[23] Tannir NM, Powles T, Motzer RJ, Analysis of regional differences in the phase 3 METEOR study of cabozantinib (cabo) versus everolimus (eve) in advanced renal cell carcinoma (RCC). Ann Oncol. 2016;27:vi285.

[24] Tannir NM, Pal SK, Atkins MB. Second-line treatment landscape for renal cell carcinoma: a comprehensive review. Oncologist. 2018;23(5):540–555. 29487224 10.1634/theoncologist.2017-0534 PMC5947457

