# Supplementary material for: Analysis by region of outcomes for patients with advanced renal cell carcinoma treated with cabozantinib or everolimus: a sub-analysis of the METEOR study
Source: Acta Oncol. Author manuscript; Available in PMC 2022 Aug 7. (PMC9357268; doi:10.1080/0284186X.2021.1995041)
Supplement: Supplementary Material [file NIHMS1825572-supplement-Supplementary_Material.docx]

**Analysis by region of outcomes for patients with advanced renal cell carcinoma treated with cabozantinib or everolimus: a sub-analysis of the METEOR study**

Manuela Schmidinger^a^, Robert J. Motzer^b^, Frederic Rolland^c^, Michael Straehler^d^, Michael Rink^e^, Margitta Retz^f^, Tibor Csoszi^g^, John A. McCaffrey^h^, Ugo De Giorgi^i^, Claudia Caserta^j^, Ignacio Duran^k^, Fawzi Benzaghou^l^, Douglas O. Clary^m^, Laurence Albiges^n^, Toni K. Choueiri^o^ and Nizar M. Tannir^p^

^a^Department of Urology, Medical University of Vienna, Austria; ^b^Memorial Sloan Kettering Cancer Center, New York, NY, USA; ^c^Institut de Cancérologie de l’Ouest, Saint-Herblain, France; ^d^Ludwig-Maximilians-Universität München, Munich, Germany; ^e^University Medical Center Hamburg-Eppendorf, Hamburg, Germany; ^f^Rechts der Isar Medical Center, Technical University of Munich, Munich, Germany; ^g^Jász-Nagykun-Szolnok County Hospital, Szolnok, Hungary; ^h^Cancer Trials Ireland, Dublin, Ireland; ^i^IRCCS Istituto Romagnolo per lo Studio dei Tumori (IRST) "Dino Amadori", Meldola, Italy; ^j^Medical and Translational Oncology Unit, Azienda Ospedaliera Santa Maria, Terni, Italy; ^k^Medical Oncology Department, Hospital Universitario Marques de Valdecilla (IDIVAL), Santander, Spain; ^l^Ipsen Bioscience, Oncology R&D, Cambridge, MA, USA; ^m^Exelixis, Inc., Alameda, CA, USA; ^n^Medical Oncology, Gustave Roussy, Université Paris-Saclay, Villejuif, France; ^o^Dana-Farber Cancer Institute, Boston, MA, USA; ^p^The University of Texas, MD Anderson Cancer Center Hospital, Houston, TX, USA

CONTACT Manuela Schmidinger, email: [manuela.schmidinger@meduniwien.ac.at](mailto:manuela.schmidinger@meduniwien.ac.at); address: Medical University of Vienna, Department of Urology and Comprehensive Cancer Centre, Währinger Gürtel 18-20, 1090 Vienna, Austria

**Supplementary material**

**Supplementary Table 1.** Best overall response in participants in the Europe and rest of the world subgroups of the METEOR study.

| **Best overall response, *n* (%)** | **Europe** | | **Rest of the world** | |
| --- | --- | --- | --- | --- |
|  | **Cabozantinib (*n =* 167)** | **Everolimus**  **(*n =* 153)** | **Cabozantinib**  **(*n =* 163)** | **Everolimus**  **(*n =* 175)** |
| Confirmed complete response (CR) | 0 | 0 | 0 | 0 |
| Confirmed partial response (PR) | 25 (15) | 6 (3.9) | 32 (20) | 5 (2.9) |
| Stable disease (SD)^a^ | 115 (69) | 99 (65) | 101 (62) | 104 (59) |
| Progressive disease (PD) | 17 (10) | 37 (24) | 24 (15) | 51 (29) |
| Not evaluable | 1 (0.6) | 2 (1.3) | 1 (0.6) | 0 |
| Missing^b^ | 9 (5.4) | 9 (5.9) | 5 (3.1) | 15 (8.6) |

^a^Patients who only have nontarget lesions and have an overall response result of non-CR/non-PD.

^b^No qualifying post-baseline assessment for overall response.

**Supplementary Table 2.** Incidence of treatment-emergent AEs in the Europe and rest of the world subgroups of the METEOR study.

| **AE, *n* (%)** | **Europe** | | **Rest of the world** | |
| --- | --- | --- | --- | --- |
|  | **Cabozantinib (*n =* 167)** | **Everolimus**  **(*n =* 151)** | **Cabozantinib**  **(*n =* 164)** | **Everolimus**  **(*n =* 171)** |
| Any AE^a^ | 167 (100) | 150 (99) | 164 (100) | 171 (100) |
| Treatment-related AE^a^ | 159 (95) | 135 (89) | 163 (99) | 158 (92) |
| Serious AE^a^ | 90 (54) | 74 (49) | 72 (44) | 80 (47) |
| Serious treatment-related AE at any time | 31 (19) | 21 (14) | 25 (15) | 20 (12) |
| AE leading to treatment discontinuation^a^ | 50 (30) | 41 (27) | 41 (25) | 54 (32) |
| AE leading to dose modification^a^ | 132 (79) | 97 (64) | 135 (82) | 107 (63) |
| Dose reduction | 106 (63) | 42 (28) | 104 (63) | 40 (23) |
| Dose hold | 119 (71) | 90 (60) | 127 (77) | 105 (61) |
| Grade 3/4 AEs^b^ | 123 (74) | 87 (58) | 113 (69) | 109 (64) |
| Anemia | 10 (6.0) | 27 (18) | 12 (7.3) | 28 (16) |
| Asthenia | 12 (7.2) | 5 (3.3) | 3 (1.8) | 3 (1.8) |
| Diarrhea | 23 (14) | 3 (2.0) | 21 (13) | 5 (2.9) |
| Fatigue | 18 (11) | 9 (6.0) | 18 (11) | 15 (8.8) |
| Hyperglycemia | 1 (0.6) | 2 (1.3) | 2 (1.2) | 14 (8.2) |
| Hypertension | 30 (18) | 10 (6.6) | 21 (13) | 2 (1.2) |
| Hypokalemia | 12 (7.2) | 4 (2.6) | 3 (1.8) | 6 (3.5) |
| Hypomagnesemia | 11 (6.6) | 0 | 5 (3.0) | 0 |
| Hyponatremia | 6 (3.6) | 4 (2.6) | 10 (6.1) | 4 (2.3) |
| PPES (hand-foot syndrome) | 16 (9.6) | 2 (1.3) | 12 (7.3) | 1 (0.6) |

^a^Includes only events that occurred within the AE observation period, which is defined as the time from the first dose date until 30 days after the date of the decision to discontinue study treatment, date of death, date of consent withdrawal, or data cutoff date, whichever occurs first.

^b^AEs occurring in at least 5% of patients in any treatment arm reported according to the National Cancer Institute Common Terminology Criteria for Adverse Events version 4.0 and listed by preferred terms coded using the Medical Dictionary for Regulatory Activities version 17.0.

AE: adverse event; PPES: palmar-plantar erythrodysesthesia syndrome.

**Supplementary Table 3.** Subsequent anticancer therapies in the Europe and rest of the world subgroups of the METEOR study.

| **Treatment, *n* (%)** | **Europe** | | **Rest of the world** | |
| --- | --- | --- | --- | --- |
|  | **Cabozantinib (*n* = 167)** | **Everolimus (*n* = 153)** | **Cabozantinib (*n* = 163)** | **Everolimus (*n* = 175)** |
| **Number of patients with at least one medication** | **64 (38)** | **69 (45)** | **63 (39)** | **86 (49)** |
| **Glucocorticoids** | **–** | **–** | **1 (0.6)** | **0** |
| Prednisone | – | – | 1 (0.6) | 0 |
| **Interferons** | **2 (1)** | **6 (4)** | **1 (0.6)** | **0** |
| Interferon alfa | 2 (1) | 6 (4) | – | – |
| Peginterferon | – | – | 1 (0.6) | 0 |
| **Interleukins** | **0** | **3 (2)** | **0** | **1 (0.6)** |
| Interleukins | 0 | 3 (2) | 0 | 1 (0.6) |
| **Monoclonal antibodies** | **0** | **2 (1)** | **6 (4)** | **6 (3)** |
| Bevacizumab | 0 | 2 (1) | 5 (3) | 6 (3) |
| Monoclonal antibodies | – | – | 1 (0.6) | 0 |
| **Nitrogen mustard analogues** | **0** | **1 (0.7)** | **1 (0.6)** | **0** |
| Cyclophosphamide | 0 | 1 (0.7) | – | – |
| Melphalan | – | – | 1 (0.6) | 0 |
| **Other cytotoxic antibiotics** | **–** | **–** | **1 (0.6)** | **0** |
| Ixabepilone | – | – | 1 (0.6) | 0 |
| **Other therapeutic products** | **1 (0.6)** | **1 (0.7)** | **6 (4)** | **9 (5)** |
| Investigational drug | 1 (0.6) | 1 (0.7) | 6 (4) | 9 (5) |
| **Platinum compounds** | **1 (0.6)** | **0** | **0** | **1 (0.6)** |
| Carboplatin | 1 (0.6) | 0 | – | – |
| Cisplatin | – | – | 0 | 1 (0.6) |
| **Protein kinase inhibitors** | **28 (17)** | **61 (40)** | **26 (16)** | **72 (41)** |
| Axitinib | 18 (11) | 35 (23) | 18 (11) | 40 (23) |
| Cabozantinib | 0 | 1 (0.7) | 0 | 4 (2) |
| Pazopanib | 2 (1) | 8 (5) | 1 (0.6) | 9 (5) |
| Sorafenib | 3 (2) | 16 (10) | 2 (1) | 7 (4) |
| Sunitinib | 4 (2) | 9 (6) | 6 (4) | 14 (8) |
| Temsirolimus | 2 (1) | 2 (1) | 3 (2) | 2 (1) |
| **Pyrimidine analogues** | **1 (0.6)** | **0** | **4 (2)** | **3 (2)** |
| Capecitabine | 1 (0.6) | 0 | – | – |
| Fluorouracil | – | – | 3 (2) | 3 (2) |
| Gemcitabine | 1 (0.6) | 0 | 4 (2) | 3 (2) |
| **Selective immunosuppressants** | **38 (23)** | **5 (3)** | **38 (23)** | **7 (4)** |
| Everolimus | 38 (23) | 5 (3) | 38 (23) | 7 (4) |
| **Vinca alkaloids and analogues** | **1 (0.6)** | **0** | **0** | **1 (0.6)** |
| Vinblastine | 1 (0.6) | 0 | 0 | 1 (0.6) |
